# Medical Opinion and Movement

**Published:** 1909-05-08

**Authors:** 


### Medical Opinion and Movement.

THE pernicious effect upon the Eyes of prolonged work with Artificial Light of too great intensity is discussed in a paper by Dr. Terrien in the *Journal de Médecine Interne*. He finds that the incandescent gas light and the electric light are liable to produce erythropsy, photophobia, and even temporary abolition of vision. These effects he attributes to the action of the ultra-violet rays upon the eye. He divides the different artificial lights into three classes, according to the proportion of ultra-violet rays they transmit. The first class comprises the candle and oil lamp, and are very poor in actinic rays. The second class includes petrol and gas (except with incandescent mantles); and the third class comprises all varieties of incandescent lamps, acetylene, and electric. From a hygienic point of view for the eye the oil lamp is by far the most suitable, but the author thinks that means can be adopted to annul the evil effects of the more modern forms of artificial light. He proposes that the electric bulbs should be tinted a light yellow, and suggests that slightly yellow-tinted glasses mounted in pince-nez or spectacles should also be worn by those who are compelled to do prolonged near work by these artificial lights.

---

AT the recent meeting of the German Surgical Congress, to which reference has already been made, the Surgery of the Ureters was fully dealt with by Garré, of Bonn, and he discussed the various methods of treating a ureter when divided in the course of an operation for removal of a malignant growth of the uterus and its appendages. In cases of reunion, the chief danger is stenosis, and on this account transverse section or suturing are especially to be avoided. If resection of a portion of the ureter is necessary, it can be stretched to the extent of two or three centimetres, and a few more centimetres are obtainable by disengaging the kidney and lowering it. Near the bladder suturing of the ureter is not possible, and the method of implantation into the bladder wall, as proposed by Novard, is usually resorted to. Failing this there remains the implantation of the ureter into the intestine or the formation of an external fistula. Intestinal implantation involves the danger of renal infection, but this is considerably reduced by making an entero-anastomosis and fixing the ureter into the isolated loop. Attempts have been made to form artificial ureters from pieces of veins, double folds of peritoneum, and caoutchouc tubes. Israel, of Berlin, has succeeded with this last-named device in two cases. One patient continued well for some years, but has since gone to Africa and not been heard of. The second patient, a young boy, remained well for a year, but then developed a pyelonephritis and died. Many other interesting subjects were brought up for discussion, and among them may be also mentioned the treatment of elephantiasis of the limbs by placing threads of silk in the subcutaneous tissue throughout the length of the limb to act in the manner of drain pipes. Brandt, of Konigsberg, reports a case successfully treated by him in this way, but no mention of Mr. Handley's name appears to have been made.

---

SOME years ago the treatment of Tuberculous Peritonitis by intra-abdominal injections of Oxygen was introduced by Thiriar, and has since been advocated by other authorities. Professor Bainbridge and Dr. Mecker, of New York, have recently adopted the introduction of oxygen into the abdominal cavity after laparotomy. The oxygen must be pure, and is sterilised and heated to a temperature of 32°-38° C. It is introduced by means of a sort of catheter at one end of the wound, after the rest of the incision has been brought together by sutures. After removal of a large tumour or the evacuation of fluid, sufficient gas is introduced so that the abdomen resumes its original size. In other cases the oxygen is allowed to distend the abdomen till the liver dulness has disappeared. The oxygen is absorbed in 24 to 36 hours. The effects of these injections of oxygen were first studied in animals by these authors and also by Professor Burkhardt, of Wurzburg. They are said to stimulate respiration, the heart, and intestinal peristalsis. They prevent the formation of adhesions, and they ensure a rapid recovery from the anæsthetic. They produce no ill effects if carried out with care and under moderate pressure. The objects are to combat shock, hæmorrhage, nausea, and post-anæsthetic vomiting; to compensate diminished abdominal pressure after removal of a tumour or fluid; and to prevent adhesions. Altogether 35 cases have been treated in this way with encouraging results. Among them there were three cases of general peritonitis, and all of them recovered.

---

THE medical treatment of Ulcer of the Stomach was fully discussed at the German Medical Congress, and some divergence of opinion was displayed by those who took part in the debate. Dr. Lenhartz opened the discussion, and gave an exposition of his method of treatment. He advocates a moderate diet for these patients from the first day, even in cases of hæmorrhage, instead of what he calls the inanition cure of Von Leube. Most of these patients have hyperacidity, and proteids tend to neutralise the hydrochloric acid of the stomach. He gives for the first three days two eggs and 200 grammes of milk. On the fourth day he adds sugar; on the sixth, minced meat; and on the following days rice and rusks. He never prescribes purgatives, but he administers a glycerin enema as soon as palpation shows fæcal accumulation in the right iliac fossa. He has treated in this way 295 patients, of whom 262 had suffered a recent severe hæmorrhage with a mortality of 2.3 per cent. On the other hand Von Leube, who also took part in the discussion, allows no food by the mouth for the first few days, but administers nutrient enemata, and then only gives a very sparse diet. He orders Carlsbad water and hot fomentations to the abdomen to assist the granulations of the ulcer. In cases of hæmorrhage he advises adrenalin, bismuth, and morphia and the local application of ice. Eleven years ago his published statistics were 74 per cent. cures, relief in 22 per cent., and 2.5 per cent. mortality. Since then he has treated 627 cases, of which 90 per cent. were cured, 8.5 per cent. improved, and 1 per cent. received no benefit from the treatment. None of the cases ended fatally. While several taking part in the discussion spoke in favour of one or other of the two methods, others deprecated a too precise and definite line of treatment for all cases.

---

COENEN, in a review of 35 cases of Supra-condylar Fracture of the Humerus, concludes that the typical form of this lesion, as ordinarily described, is exceedingly rare. He has only recorded one such case, produced by forcible hyper-extension, and is of opinion that it would be well worth while to collect further statistics with regard to the lesion and to revise the nomenclature of humeral fractures. Theoretically, as every student knows, a large number of varieties of fracture in the region of the elbow joint have been described, and while disregarding all lesions which do not affect the humerus itself, it is still clear that the descriptions of so-called purely supra-condylar fractures do not always correspond with the nomenclature assigned to them. In children the supra-condylar fracture must be exceedingly rare; what is usually taken for it is a separation of the condylar epiphysis. Regarding the treatment of the pure variety, as seen in adults, the author protests against the ordinarily described methods which bear the names of Græssner's extension and Hilgenreiner's angular flexion methods. Coenen prefers to treat his cases by means of an ordinary obtuse angled splint, with the arm in full supination. He lays stress on the accurate coaptation of the fragments and reduction of the fracture by traction. For this purpose he anæsthetises his patient, reduces the fracture fully, accurately corrects any angular deviation that may be present, and puts up the arm in his obtuse angled splint. This method gives excellent results.

---

GANDOLFO, in a recent number of *Il Morgagni*, reports shortly the results obtained by Mercurial Ointments in the treatment of commencing Suppurative Lesions. In England inunction is by no means so generally used as on the Continent, and nowhere, apparently, is this method of treatment more popular than in Italy. That it is a method which often proves most efficacious there can be no question; but it is one, too, that is open to some valid objections. Putting aside entirely, for the moment, the allegation which is sometimes made by English physicians that it is a "filthy" way of giving mercury, there remain the more reasonable objections that it is difficult, when the inunction method is employed, to regulate the dosage, and that the method is not a popular one with patients. This last objection is of some importance, especially in private practice, but it scarcely holds good in the case of children. In most instances inunctions will be found almost an ideal method for getting children, especially infants, under the influence of mercury. The administration of mercurial salts by mouth very often leads to obstinate gastro-intestinal derangement in the case of infants, although it must be admitted that there is far less tendency towards such derangement when small doses, frequently repeated, of hyd. cum creta are employed than when the other mercurial salts are given alone and treatment is pressed. Subcutaneous injections are certainly not advisable in the case of infants, and in the case of adults they demand more time and are far more troublesome to practitioner and patient alike than the inunction method.

---

WITH us Mercurial Inunction, when it is used, is largely limited to syphilitic cases, though its value in chronic peritonitis and some other conditions has long been well known. Gandolfo, in the contribution alluded to above, shows its extreme value in cases of pleurisy, and also praises it in other conditions. He has found it useful in glandular lesions of tubercular origin, and states that in many cases a prompt application of mercurial ointment leads to a striking improvement in incipient suppurative lesions, among which he instances phlegmonous conditions generally, appendicitis, and otitis media and mastoiditis. The cases he adduces are, however, too few to warrant the high claims he makes, except in cases of pleurisy and localised abdominal and pelvic lesions of a chronic inflammatory nature. In these the value of mercurial inunction has long been known, but it is worth while from time to time to draw attention to it.

---

A PLEA for the use of local Infiltration Anæsthesia in Tracheotomy is urgently advanced by Chevalier Jackson in the *Laryngoscope*. General anæsthesia is condemned as highly dangerous, and quite unnecessary if the method of intradermal infiltration of Schleich is used; but the author at the same time does not confirm the idea of Brown-Sequard, that if the first incision be exactly in the middle line it will anæsthetise the deeper tissues. It is pointed out that the preservation of the cough reflex is all important when the trachea is opened; for the deep inspiration which immediately follows may suck blood or pus into the bronchi, and unless at once expelled by coughing septic pneumonia may supervene. Morphine, codeine, and other sedatives are equally objectionable for the same reasons. Deliberate and careful dissection, absolute hæmostasis before opening the trachea, and full asepsis are all insisted on; and it is pointed out that to secure these operation in good time is essential; to stab at the trachea of a cyanosed patient almost *in extremis*, at the bottom of a deep pool of blood, is to run grave risk of damaging other structures, to say nothing of failing to open the trachea. Careful after-treatment is most important, and nurses should be especially trained for this work. Some infection of the wound edges by the tracheal secretions is inevitable, but is seldom troublesome whereas any failure in aseptic technique of the operation or dressing may be disastrous. Dr. Jackson makes up his anæsthetic solution with one grain of cocaine hydrochlorate and one drop of carbolic acid to an ounce of sterilised water. To boil a cocaine solution destroys its anæsthetic property.

---

Ekmekdjian has published the results of his researches into the Origin of Hysterical Hemianæsthesia, and the following are his conclusions: (1) Hemianæsthesia can be caused to appear by suggestion, and made to disappear by persuasion, and is thus identical with all the other manifestations of hysteria. (2) The presence of heminanæsthesia in a patient shows that he is pathologically subject to suggestion, and is on this account a hysterical subject, since the characteristic of hysteria is its suggestibility. (3) Its presence does not prove that all the other ailments of the same patient are necessarily of a hysterical nature; these ailments may be of a different nature and merely running their course in a hysterical subject. (4) Suggestion or auto-suggestion is the starting-point of all hemianæsthesia, but it is not always easy to prove their presence, and it is not right therefore to deny their existence. (5) The commonest sources of suggestion are the doctor's leading questions and suggestive procedures when looking for the hemianæsthesia. All questions and manœuvres likely to suggest to the patient the idea of a possible hemianæsthesia must therefore be carefully avoided. (6) Questions as to the degree of sensation produced by the pin-pricks are valueless for diagnosis.

---

The Destructive Action of the Toxins of Bacillus Pyocyaneus upon other bacteria is well known. Emmerich and Löw are of opinion that this action is due to a substance of the nature of an enzyme, which they call pyocyanase. The fact that Escherich had used it in cases of cerebro-spinal meningitis stimulated Krausz and Goosz to employ it in gonorrhœal urethritis, with, however, purely negative results. Despite this failure, Emmerich continued to urge its use in diphtheria and gonorrhœa, and further experiments were made by Sellei, who publishes his results in the *Zeitschrift für Urologie.* He took three or four day old cultures of bacillus pyocyaneus on agar, and diluted them with serum. The toxin of bacillus pyocyaneus being an endo-toxin, five days were allowed to elapse before killing the organisms in the emulsion by heat. A pale green liquid was thus obtained, and on mixing this with discharges containing gonococci, it was found impossible to make cultures of the latter. After repeating this experiment several times, Sellei made others with bouillon-cultures of bacillus pyocyaneus. At 60° F. these cultures had no effect on the growth of the gonococcus, whereas at 98° they had a distinct though variable bactericidal influence. As is so often the case, however, the results *in vitro* are not borne out by those obtained in actual practice, the anatomical peculiarities of the urethra preventing the bactericidal substances coming into sufficiently close quarters with the offending organisms.

---

Zweifel, in the *Therapeutische Monatshefte,* describes a uterine condition which he terms Septic Constriction of the Uterus. This is a kind of "uterine tetanus," which persists with great violence during parturition, and gives rise to such an energetic gripping of the fœtus or placenta that they are expelled with the greatest difficulty, if at all. The author considers that this complication is much more frequent than is generally admitted, its presence being frequently overlooked, especially in cases of contracted pelvis. When diagnosed its existence is attributed to the premature exhibition of ergot or to intra-uterine injury—*e.g.* by attempts at version. While admitting the possible influence of ergot and trauma, the author has noted in all cases under his own care, and in which there could be no question of these factors being at work, that the contents of the uterus are macerated and the membranes prematurely ruptured some hours before delivery. The slow and insidious flow of the liquor amnii occurring a long time before normal evacuation of the uterus allows of an ascending infection of the genital tract. The alkaline fluid from the ovum neutralises the acidity of the vaginal secretions, which thus become favourable for the multiplication of germs. Streptococci, staphylococci, bacillus coli, and pyogenic anærobic bacilli have been found in such uteri. To avoid such grave complications in cases in which the amniotic fluid has escaped, the author, while insisting on the necessity for rapid delivery of the fœtus, advises vaginal douches of artificial serum containing .5 per cent. of lactic acid, a solution which corresponds chemically to the non-bactericidal vaginal secretions of pregnant women.

---

Caussade and Leven have contributed a paper to the Société Médicale des Hôpitaux on the subject of Changes in Body Weight in the course of Jaundice due to retention of bile. Most authors affirm that wasting inevitably follows this type of jaundice because the fat in the food is undigested and unabsorbed. Caussade and Leven, on the contrary, maintain that retention of bile is not in itself sufficient to account for loss of weight in jaundice, since in their clinic at the Hôpital Tenon they have noticed many cases in which the body weight remained stationary, or even increased with careful dieting in bed. The authors give milk in three-hourly feeds of 350 grams, flavoured to taste with tea, coffee, vanilla, or sugar, and given hot or cold as preferred by the patient. If under this system of forced feeding the patient continues to lose weight, they assume that the cause of the icterus is malignant, whereas if the weight remains stationary the obstruction is mechanical merely. This general statement must, of course, be modified in cases in which œdema, ascites, increase in size of the liver, etc., will account for an increase in weight, as also in cases where tuberculosis, diabetes, or other wasting disease can be held responsible for any emaciation.

